# Case Report: Multiple Seizures After a Diphenoxylate-Atropine Overdose in a Small Child

**DOI:** 10.3389/fphar.2021.646530

**Published:** 2021-04-12

**Authors:** Chun-Hui Wang, Li-Jia Song, Yang Yang, Xiao-Peng Qu, Li Lan, Bei Liu

**Affiliations:** ^1^ Department of Pediatrics, Tangdu Hospital, Air Force Medical University, Xi’an, China; ^2^ Department of Neurosurgery, Tangdu Hospital, Air Force Medical University, Xi’an, China

**Keywords:** children, toxic encephalopathy, seizure, neuropharmacology, diphenoxylate-atropine

## Abstract

Poisoning is a type of accidental injury and it is considered a major public health problem worldwide. Oral drug poisoning in children is an important cause of accidental injury and even death. It is a common critical emergency in the field of pediatrics. Once a child unintentionally takes an overdose, regardless of whether it caused poisoning or not, they should be admitted to the hospital for emergency treatment. Acute poisoning in children most frequently occurs through the digestive tract. Drug poisoning can happen in children of all ages. In children younger than 1 year, drug poisoning is mostly caused by the parents during feeding, while in children aged 1–3 years, it predominantly occurs as a result of an accident. A case of diagnosis and treatment of a child with diphenoxylate-atropine poisoning is reported herein. The early manifestation of this child was acute toxic encephalopathy with clinical manifestations of a coma, convulsions, and respiratory depression. A brain MRI showed extensive damage to the bilateral caudate nucleus, lenticular nucleus, parietal lobe, precuneus lobe, and occipital lobe. Accidental administration of a large dose of diphenoxylate results in severe clinical symptoms and can cause obvious diffuse brain damage.

## Introduction

Diphenoxylate, also known as penehyclidine and cyanophenoxylate, is used in the treatment of acute and chronic functional diarrhea and chronic enteritis. The conventional therapeutic amount of this drug has no effect on the central nervous system (Murphy et al., 2009). However, diphenoxylate poisoning caused by improper dosage is relatively common, particularly in the population of children under 10-years-old. At doses significantly higher than the therapeutic amount, the occurrence of strong respiratory depression and coma is observed (Karim et al., 1972). Thus, diphenoxylate has been listed as one of the major harmful drugs in pediatrics (Shee and Pounder, 1980; Thomas et al., 2008). Even low doses of diphenoxylate can cause poisoning in children; therefore, it is not suitable for the treatment of diarrhea as a conventional drug. Nonetheless, some doctors may lack understanding of the side effects caused by the drug, which might lead to overdose and poisoning. Moreover, many families use diphenoxylate as a home remedy for diarrhea. The drug is available in the form of sugar-coated tablets; thus, improper storage often leads to serious symptoms of poisoning in infants and young children.

### Case Presentation

A 3-year-old boy accidentally swallowed diphenoxylate-atropine on May 12, 2019. The quantity of the ingested drug was 35 tablets (each tablet contained diphenoxylate 2.5 mg and atropine 0.025 mg), which was confirmed by the parents. The patient experienced a respiratory arrest on the way to the hospital and recovered after cardiopulmonary resuscitation in the ambulance. In the pediatric emergency department, warm saline water was administered for gastric lavage until the liquid was clear, and the gastric tube was retained. Liquid paraffin was injected through the gastric tube for catharsis. Concurrently, warm saline solution and liquid paraffin enema were given to facilitate the discharge of diphenoxylate. Naloxone was administered to excite the respiratory center to fight against the respiratory depression caused by diphenoxylate overdose. On the second day of admission, the child was conscious and fluent in response to questions. However, on the third day of admission, the child suddenly experienced frequent generalized tonic-clonic seizures (GTCS) and subsequently developed status epilepticus. Midazolam was given as a continuous intravenous infusion of 10 μg/kg/min to control the seizures.

**FIGURE 1:**
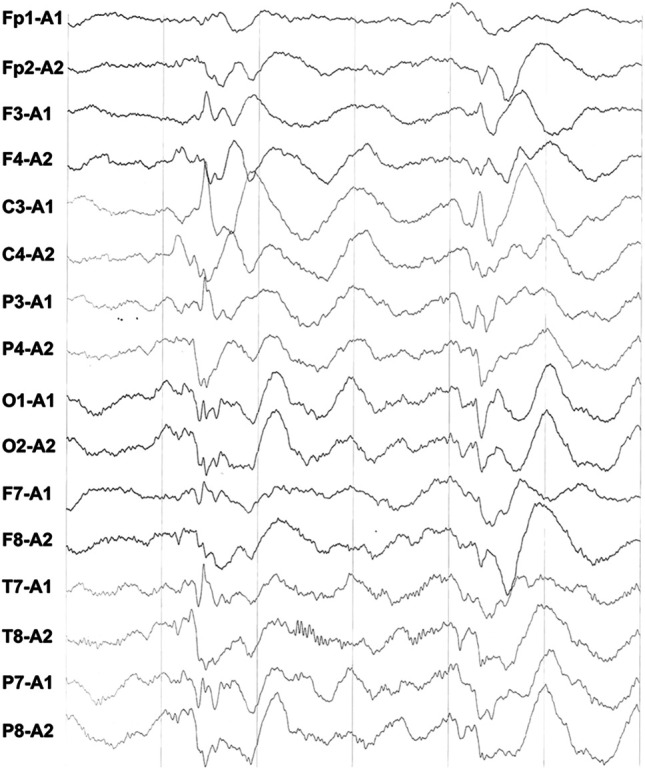
During interictal EEG, diffuse irregular high amplitude slow waves of bilateral symmetry were found.

**FIGURE 2:**
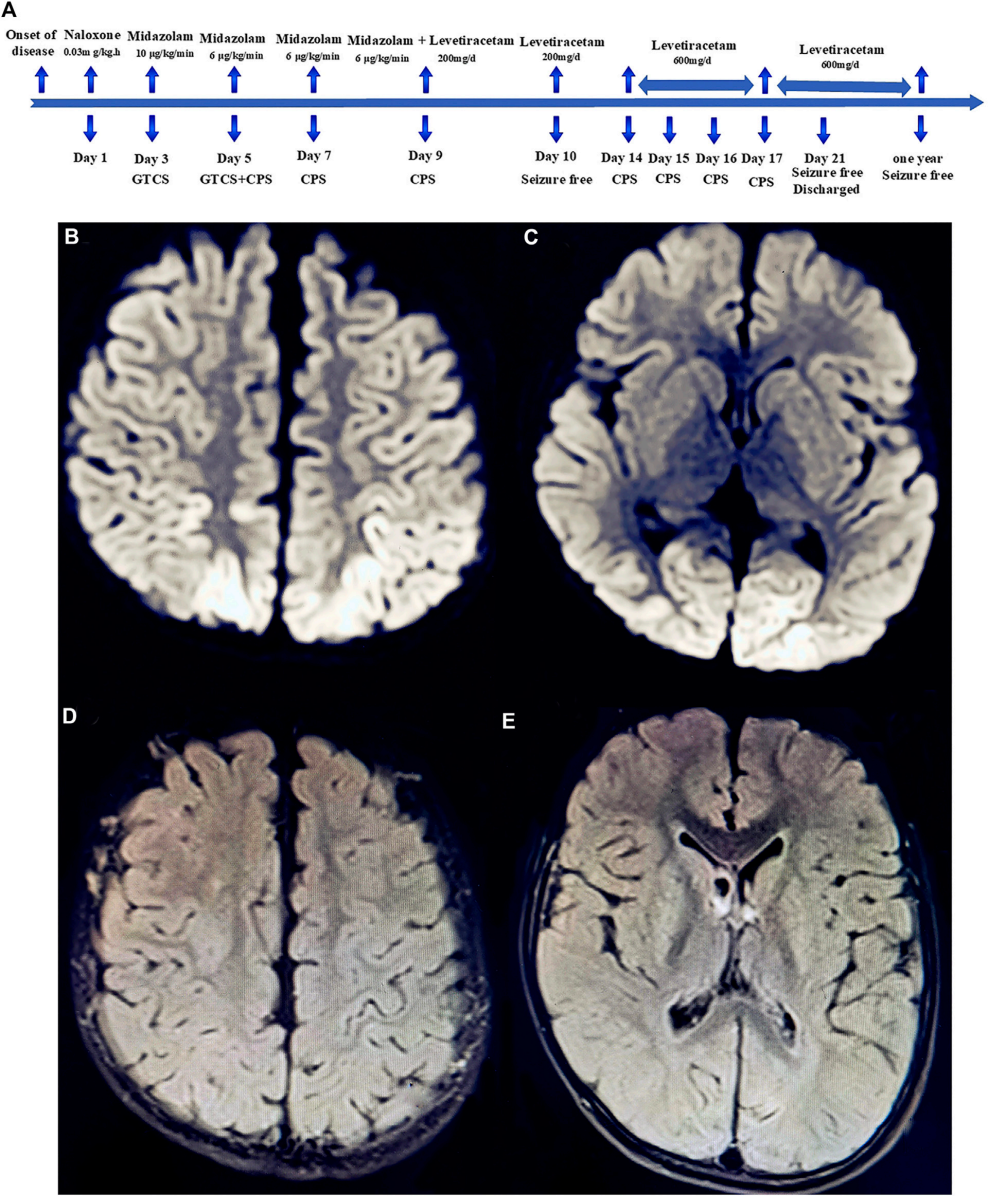
Timeline of the patient’s clinical course and outcome **(A)**. Diffusion-weighted magnetic resonance imaging (DW-MRI) of the head showed abnormal high signal intensity in the bilateral parietal lobe **(B)**, anterior wedge, and occipital lobe **(C)**. Head MRI-DWI re-examined after 1 year of follow-up revealed that the abnormal signal intensity of brain gray matter disappeared completely **(D,E)**.

The body temperature was 37.7°C, and all blood results were within normal limits, except for the percentage of neutrophils (89.3% with the reference range of 31–40%). EEG showed numerous irregular high amplitude slow waves and sharp slow waves during the interictal period (Figure 1). No abnormalities were found in the head CT. The convulsions were difficult to manage; however, gradual control was achieved following combined use of a levetiracetam oral liquid. The diffusion-weighted imaging (DWI) of the head MRI revealed that the bilateral parietal lobe, precuneus, and occipital lobe exhibited brain-like hyperintensity, with apparent diffusion coefficient (ADC) values of 0.000644–0.000811 (Figures 2B,C).

On the fifth day of admission, the child developed complex partial seizures (CPS), with specific manifestations as follows: loss of consciousness, upward gaze, frequent blinking lasting for approximately 8–10 s, no limb tonic-clonic twitch, seizure frequency 2–4 times per day. Following the administration of a levetiracetam oral solution, the frequency of seizures was considerably reduced. CPS disappeared on the 10th day of admission, but reappeared on the 14th day. After 3 weeks of treatment, the child showed substantial clinical improvement. The boy was discharged from the hospital and took oral levetiracetam to prevent seizures. At a 12-months follow-up, the child felt good and experienced no seizures (Figure 2A). For the administration schedule, see Table 1. No abnormality was found in MRI-DWI (Figures 2D,E).

**TABLE 1:** Clinical features, treatment options, and outcome in this child.

Period	Symptoms and events	Treatment options
May 12, 2019, 23:00	Unconsciousness, cyanosis of the lips, shallow breathing, heart rate 10–20 beats/min, vomiting once before the onset of illness, the patient’s mother discovered that he had taken 35 diphenoxylate-atropine tablets by mistake	Cardiopulmonary resuscitation immediately in the ambulance, naloxone 2 mg was injected intravenously
First day of admission, May 13, 2019	Pauses for 2 times during transfer. On the first physical examination in hospital, he was in a coma, blood pressure 60–84/32–44 mmhg, weak and shallow breathing, bilateral pupil diameter 1mm, equal circle, slow reflection of light, slightly cyanotic lips	0.9% normal saline 2,000 ml, gastric lavage; Naloxone 0.03 mg/k g·h, ivgtt. Furosemide 1.5 mg, iv; Dopamine 10 μg/kg/min to maintain blood pressure; Ventilator assisted breathing; Liquid paraffin was injected into the stomach through gastric tube for catharsis; Warm saline and liquid paraffin were used for enema to promote drug excretion
The day after admission May 14, 2019	Consciousness, fluent speech	Naloxone 0.03 mg/k g·h, ivgtt
The third day of admission May 15, 2019	Frequent GTCS	Chloral hydrate 8 ml, enema; Midazolam 6.5 mg, iv; Midazolam 10 μg/kg/min, ivgtt; Phenobarbital sodium, 27 mg, im, tid
The fourth day of admission May 16, 2019	GTCS	Midazolam 6 μg/kg/min, ivgtt; Phenobarbital sodium, 27 mg, im, tid
The fifth day of admission May 17, 2019	GTCS + CPS	Midazolam 6 μg/kg/min, ivgtt; Phenobarbital sodium, 27 mg, im, tid
The sixth day of admission May 18, 2019	GTCS + CPS	Midazolam 8 μg/kg/min, ivgtt
The seventh day of admission May 19, 2019	CPS	Midazolam 6 μg/kg/min, ivgtt
The eighth day of admission May 20, 2019	CPS	Midazolam 6 μg/kg/min, ivgtt
The ninth day of admission May 21, 2019	CPS	Midazolam 6 μg/kg/min, ivgtt; Levetiracetam, 100 mg, oral, bid
The 10th day of admission May 22, 2019	Seizure free	Levetiracetam, 200 mg, oral, bid
The 14th day of admission May 26, 2019	CPS	Levetiracetam, 300 mg, oral, bid
The 15th day of admission May 27, 2019	CPS	Levetiracetam, 300 mg, oral, bid; Clonazepam, 0.5 mg, oral, qd
The 16th day of admission May 28, 2019	CPS	Levetiracetam, 300 mg, oral, bid; Clonazepam, 0.5 mg, oral, bid
The 17th day of admission May 29, 2019	CPS	Levetiracetam, 300 mg, oral, bid; Clonazepam, 0.5 mg, oral, bid
The 17th day of admission May 30, 2019	Seizure free	Levetiracetam, 300 mg, oral, bid; Clonazepam, 0.5 mg, oral, bid
The 21st day of admission June 02, 2019	Seizure free, Discharged	Levetiracetam, 300 mg, oral, bid
The third month after discharge	Seizure free	Levetiracetam, 300 mg, oral, bid
One year after discharge	Seizure free	Levetiracetam, 300 mg, oral, bid

GTCS, generalized tonic-clonic seizure; CPS, complex partial seizures.

## Discussion

A diphenoxylate-atropine overdose in children can lead to poisoning, which might be related to various physiological characteristics. On the one hand, the function of the blood-brain barrier in children is imperfect. Thus, the drug can easily produce an inhibitory effect on the central nervous system by passing through the blood-brain barrier. On the other hand, the liver function in children is immature; therefore, drug metabolism is slow and diphenoxylate can remain in the body for a long time.

Although the studied child underwent two gastric lavages, some of diphenoxylate-atropine was discharged into the intestines due to the excessive ingestion of the drug. Gastric lavage and catharsis did not lead to complete excretion. Consequently, diphenoxylate-atropine was absorbed in the intestines, which led to brain damage and seizures.

Anoxic-ischemic encephalopathy caused by diphenoxylate-atropine is the most likely diagnosis. There were some early discussions regarding the likely diagnosis. Due to multiple epileptic seizures and fever, the child was at first suspected to suffer from a central nervous system infection; however, this diagnosis was excluded following cerebrospinal fluid testing and continuous monitoring of the body temperature.

Diphenoxylate is one of the deadly single dose agents, which can cause accidental home poisoning in children (Euwema and Swanson, 2020). In the past, 10 children with acute diphenoxylate-atropine poisoning were admitted to our department. Nonetheless, the clinical manifestations of poisoning were predominantly mild and mainly included gastrointestinal symptoms. The degree of respiratory depression was minor. The clinical symptoms of this case were severe and could be related to the overdose ingestion of diphenoxylate-atropine. Diphenoxylate prolongs the transit time of the stomach and small intestine contents, and the drug absorption delay might cause secondary poisoning (Xiao et al., 2011). The characteristics of the MRI in the late stage were consistent with the manifestations of poisoning-induced hypoxic ischemic encephalopathy, and the degree of MRI lesions was highly correlated with clinical symptoms.

We have the following experience in the treatment of children with diphenoxylate poisoning. First, children suffering from diphenoxylate poisoning should be given regular gastric lavage because absorption might be delayed secondary to the inhibition of the gastrointestinal motility (Michael and Sztajnkrycer, 2004). A review by Thomas et al. demonstrated that diphenoxylate-atropine poisoning can cause serious complications, such as aspiration pneumonia, cerebral edema, and death (Thomas et al., 2008). In some cases, intact pill fragments were found 15–27 h after ingestion (Rumack and Temple, 1974; Wasserman et al., 1975; Thomas et al., 2008). Thus, once children accidentally swallow excessive amounts of diphenoxylate-atropine, gastric lavage and catharsis are required as soon as possible. In this case, after thorough gastric lavage, a gastric tube was inserted and liquid paraffin was used for catharsis. Second, timely administration of intravenous rehydration and diuretic drugs promotes the excretion of diphenoxylate. Third, the rescue drug for diphenoxylate poisoning is naloxone (McCarron et al., 1991). Early and appropriate continuous intravenous application of naloxone is the key to successful treatment of diphenoxylate poisoning. Children with severe poisoning were given naloxone 0.05–0.1 mg/kg. After the first injection, administration by intravenous drip infusion was performed at a rate of 10–20 μg/(kgh) for 2–3 days. The drug dosage was gradually reduced when the breathing was stable and the mind appeared clear. Administration of naloxone in children with severe poisoning should not be stopped too early. The drug should be administered repeatedly to avoid recurrence of respiratory depression. Finally, excessive intake of diphenoxylate might cause toxic encephalopathy; therefore, prophylactic administration of antiepileptic agents should be considered.

## Conclusion

Overdose diphenoxylate-atropine ingestion often leads to secondary absorption in the intestines, which causes brain damage. Continuous intravenous administration of naloxone is necessary for severely poisoned children. Early gastric lavage and repeated catharsis are key to reducing impairment and disability. Naloxone should not be stopped early in the treatment of children with severe diphenoxylate-atropine poisoning. Safe storage of toxic drugs is an effective way to prevent accidental swallowing by children.

## Data Availability

The original contributions presented in the study are included in the article/Supplementary Material, further inquiries can be directed to the corresponding authors.
